# Effect of genomic selection and genotyping strategy on estimation of variance components in animal models using different relationship matrices

**DOI:** 10.1186/s12711-020-00550-w

**Published:** 2020-06-11

**Authors:** Lei Wang, Luc L. Janss, Per Madsen, John Henshall, Chyong-Huoy Huang, Danye Marois, Setegn Alemu, AC Sørensen, Just Jensen

**Affiliations:** 1 Center for Quantitative Genetics and Genomics, Aarhus University, Aarhus, Denmark; 2 Cobb-Vantress Inc, Siloam Springs, USA

**Keywords:** Variance components, Animal model, Single-step GBLUP, Genotyping strategy, Bias

## Abstract

**Background:**

The traditional way to estimate variance components (VC) is based on the animal model using a pedigree-based relationship matrix (**A**) (A-AM). After genomic selection was introduced into breeding programs, it was anticipated that VC estimates from A-AM would be biased because the effect of selection based on genomic information is not captured. The single-step method (H-AM), which uses an **H** matrix as (co)variance matrix, can be used as an alternative to estimate VC. Here, we compared VC estimates from A-AM and H-AM and investigated the effect of genomic selection, genotyping strategy and genotyping proportion on the estimation of VC from the two methods, by analyzing a dataset from a commercial broiler line and a simulated dataset that mimicked the broiler population.

**Results:**

VC estimates from H-AM were severely overestimated with a high proportion of selective genotyping, and overestimation increased as proportion of genotyping increased in the analysis of both commercial and simulated data. This bias in H-AM estimates arises when selective genotyping is used to construct the **H**-matrix, regardless of whether selective genotyping is applied or not in the selection process. For simulated populations under genomic selection, estimates of genetic variance from A-AM were also significantly overestimated when the effect of genomic selection was strong. Our results suggest that VC estimates from H-AM under random genotyping have the expected values. Predicted breeding values from H-AM were inflated when VC estimates were biased, and inflation differed between genotyped and ungenotyped animals, which can lead to suboptimal selection decisions.

**Conclusions:**

We conclude that VC estimates from H-AM are biased with selective genotyping, but are close to expected values with random genotyping.VC estimates from A-AM in populations under genomic selection are also biased but to a much lesser degree. Therefore, we recommend the use of H-AM with random genotyping to estimate VC for populations under genomic selection. Our results indicate that it is still possible to use selective genotyping in selection, but then VC estimation should avoid the use of genotypes from one side only of the distribution of phenotypes. Hence, a dual genotyping strategy may be needed to address both selection and VC estimation.

## Background

Accurate and unbiased prediction of breeding values (BV) is essential for an efficient breeding program to select parents of future offspring and maximize genetic gain. The linear mixed animal model [1] is commonly used for predicting BV. The distribution of BV is assumed to follow a multivariate normal distribution with a null mean and a (co)variance matrix, which is the product of two parts, a relationship matrix and the genetic variance. The numerator relationship matrix (**A**) based on pedigree is widely used as the relationship matrix in the animal model, and in this paper, the animal model that uses the **A** matrix as (co)variance matrix for BV is denoted as A-AM. To predict BV from A-AM, variance components (VC) including genetic variance and residual variance are needed to solve the mixed model equations (MME). In many cases, VC need to be estimated from the available data. If a population is under selection based on phenotype and pedigree information, if all the data that drive selection decisions are included in the model, and if the genetic architecture of the trait is close enough to the assumption of an infinite number of loci contributing to the trait, VC estimated by restricted maximum likelihood (REML) from A-AM are unbiased [2].

During the last decade, affordable high-throughput genotyping products for livestock have enabled breeding companies to use dense markers such as single nucleotide polymorphisms (SNPs) to aid prediction of genetic merit and select superior breeding stock [3–5]. Instead of using the pedigree-based relationship matrix ${\mathbf{A}}$, a genomic relationship matrix (${\mathbf{G}}$) is constructed as a proposed measure of the (co)variance of BV between genotyped animals [6]. The animal model was then modified to use ${\mathbf{G}}$ as the (co)variance matrix for BV [7]. However, due to the cost of genotyping on available platforms, not all animals in the breeding populations can be genotyped. Thus, to accommodate for partial genotyping, the so-called single-step method was developed [8–10]. The single-step method is based on a relationship matrix (${\mathbf{H}}$), which is constructed by combining ${\mathbf{G}}$ and ${\mathbf{A}}$. In this paper, the single-step method was abbreviated as H-AM to emphasize that H-AM remains an animal model but with a (co)variance structure that differs from that of A-AM. H-AM allows the simultaneous estimation of BV for all individuals when only a subset of the animals are genotyped [11].

In many livestock breeding programs, H-AM is now used for genomic evaluation [5, 11, 12], but VC are still estimated by REML from A-AM without including genomic information. For such populations under intensive genomic selection, one concern is that genomic selection may have an effect on estimating VC from A-AM. We anticipate that VC estimates from A-AM could be biased after introduction of genomic selection, because A-AM can only account for phenotype and pedigree-based selection, but not for genomic selection. Consequently, H-AM can be the natural choice for estimating VC in populations in which H-AM has been implemented for genetic evaluation. To our knowledge, there are no studies that have investigated whether, for populations under genomic selection, H-AM is a better model to estimate VC, and whether A-AM gives biased estimates of VC.

If we use H-AM to estimate VC, another concern is the effect of the genotyping strategy on bias in VC. Selective genotyping has been reported to lead to bias in prediction of BV [13], and we hypothesized that the same could be true for the estimation of VC. In most breeding programs, the genotyping strategy aims mainly at maximizing genetic gain while limiting genotyping cost. To achieve this goal, many studies showed that, for a fixed genotyping budget, selective genotyping based on own performance, results in maximum genetic response [14–16]. However, these studies assumed that VC were known. In addition, in many practical cases so far investigated, the number of genotyped individuals is small compared to the size of the whole population, so it is difficult to detect the influence of genotyping strategy on the estimation of VC. In broiler breeding the number of breeding candidates that are likely to be genotyped is relatively large, and genomic selection has already been used for several generations. If bias exists in VC estimates due to the genotyping strategy, broiler populations are ideal to detect it. So far, no studies have investigated whether bias in BV due to selective genotying, as reported in [13], also occurs in the estimation of VC, and whether the proportion of genotyped individuals impacts the amount of bias.

In this paper, we report VC estimated by A-AM and H-AM by using a commercial broiler dataset for which a large number of individuals are genotyped. The first and main objective of this study was to report, backed-up by a simulation study, that H-AM provides (highly) overestimated genetic variances when genotyping is selective and a sufficiently large proportion of individuals is genotyped. The secondary objectives were to investigate whether A-AM, or H-AM with random genotyping, could provide unbiased estimates of VC, and how genotyping strategy, proportion of genotyping and VC estimates affect predicted BV.

## Methods

### Relationship matrices for A-AM and H-AM

The relationship matrix based on pedigree is denoted ${\mathbf{A}}$ and is used in A-AM. For genotyped animals, the genomic relationship matrix ${\mathbf{G}}$ was computed by VanRaden’s method I [6], with ${\mathbf{G}} = \left( {{\mathbf{M}} - 2{\mathbf{p}}{\mathbf{1}}^{\prime}} \right)\left( {{\mathbf{M}} - 2{\mathbf{p}}{\mathbf{1}}^{\prime}} \right)^{'} /\mathop \sum \limits_{\text{j}} 2{\text{p}}_{\text{j}} \left( {1 - {\text{p}}_{\text{j}} } \right)$, where ${\mathbf{M}}$ is the matrix of genotypes with one row for each individual and one column for each SNP, and genotypes are coded as 0, 1, 2, ${\mathbf{p}}$ is the vector of allele frequencies computed from genotyped animals, and **1** is a vector of ones.

The difference between H-AM and A-AM is the (co)variance structure for BV, where the ${\mathbf{A}}$ matrix is replaced by a ${\mathbf{H}}$ matrix. To compute ${\mathbf{H}}$, first, the values in the genomic relationship matrix are aligned to the pedigree relationship matrix for genotyped individuals ${\mathbf{A}}_{22}$ by calculating ${\mathbf{G}}_{{\mathbf{a}}} =\upalpha\; + \;\upbeta{\mathbf{G}}$, where $\upalpha$ and $\upbeta$ are computed by solving the two following equations [11]:

$${\text{avg}}\left( {{\text{diag}}\left( {{\mathbf{A}}_{22} } \right)} \right) = \;\upalpha + {\text{avg}}\left( {{\text{diag}}\left( {\mathbf{G}} \right)} \right)\upbeta ,$$

and

$${\text{avg}}\left( {{\mathbf{A}}_{22} } \right) =\upalpha + {\text{avg}}\left( {\mathbf{G}} \right)\upbeta ,$$

where ${\text{avg}}()$ is a function to calculate the numeric average of all the elements in a matrix, and ${\text{diag}}()$ represents the diagonal elements of a matrix. Second, we weighted the aligned ${\mathbf{G}}_{\text{a}}$ with a small portion of pedigree relationships to obtain a genomic relationship matrix ${\mathbf{G}}^{*} = \left( {{\mathbf{1}} - {\text{w}}} \right){\mathbf{G}}_{{\mathbf{a}}} + {\text{w}}{\mathbf{A}}_{22}$. In all our analyses, the weight on the pedigree was w = 0.05. Then, ${\mathbf{G}}^{*}$ is used in the computation of the ${\mathbf{H}}$-inverse matrix as [10, 17]:

$${\mathbf{H}}^{ - 1} = {\mathbf{A}}^{ - 1} \; + \;\left( {\begin{array}{*{20}c} {\mathbf{0}} & {\mathbf{0}} \\ {\mathbf{0}} & {{\mathbf{G}}^{* - 1} \; - \;{\mathbf{A}}_{22}^{ - 1} } \\ \end{array} } \right),$$.

where ${\mathbf{A}}_{22}^{ - 1}$ is the inverse of the part of the pedigree relationship matrix for genotyped animals.

### Commercial broiler data

The commercial broiler data were provided by Cobb-Vantress Inc. (Siloam Springs, AR, USA), and the trait was body weight (BW) measured in grams at a fixed age after birds were hatched. This purebred broiler line was first selected for several selection rounds (SR) based on predicted BV from A-AM, followed by a number of SR based on predicted BV from H-AM. Table 1 provides an overview of the data. The pedigree consisted of 128,004 birds, phenotypic records were collected on 108,555 birds, and genotypes were available for 23,688 birds. Among the genotyped birds, 58 birds had no BW records. The pedigree included 53,855 males and 54,700 females with BW records, among which 8850 males and 14,848 females were genotyped. The pedigree contained 944 sires and 4113 dams, among which 76 sires and 674 dams had their own BW records, and 73 sires and 672 dams were genotyped, i.e. almost all the parents with phenotypic records were genotyped. Birds were genotyped with a 60 K SNP panel [18]. Quality control is described in [12]. After editing, 43,517 autosomal SNPs remained to compute the ${\mathbf{G}}$ matrix.

**Table 1:** Overview of the commercial broiler data

Numbers of birds	SR	Total	Males	Females	Sires	Dams
In the pedigree	1–77	128,004			944	4113
With a phenotype	68–77	108,555	53,855	54,700	76	674
With a genotype	68–77	23,688	8850	14,838	73	672

*SR* selection round

### Analysis model

For the commercial dataset, the genotyping strategy and proportion of genotyping differed between males and females (see below). Therefore, part of the strategy used to investigate the effects of genotyping strategy and proportion of genotyping compared analyses from the whole dataset with analyses for each sex. The model for this analysis is as follows:

$${\mathbf{y}} = {\mathbf{Xb}} + {\mathbf{Wm}} + {\mathbf{Zu}} + {\mathbf{e}},$$.

where ${\mathbf{y}}$ is a vector of BW (for the whole dataset or for one sex only), ${\mathbf{b}}$ is a vector of fixed effects for the contemporary group, and for sex when analysing all the data, ${\mathbf{m}}$ is a vector of random maternal permanent environmental effects, ${\mathbf{u}}$ is a vector of BV, ${\mathbf{e}}$ is a vector of residuals. ${\mathbf{X}}$, ${\mathbf{W}}$, and ${\mathbf{Z}}$ are the design matrices for fixed effects, maternal permanent environmental effects, and additive BV, respectively. For A-AM and H-AM, the covariance structures of ${\mathbf{u}}$ are ${\mathbf{A}}$ and ${\mathbf{H}}$, respectively, and those of ${\mathbf{m}}$ and ${\mathbf{e}}$ are identity matrices. VC were estimated by AI-REML using either A-AM or H-AM using the DMU package [19]. Fixed and random effects were estimated/predicted by best linear unbiased estimators (BLUE) and best linear unbiased predictors (BLUP) from A-AM or H-AM.

### Effects of genotyping strategy and proportion of genotyping in the commercial data

For the commercial data, we studied the effects of genotyping strategy and proportion of genotyping on VC estimates from A-AM and H-AM by using different subsets of the dataset.

In the first set of analyses, we compared VC estimates from A-AM and H-AM for the whole dataset, and for males and females separately, and linked differences in VC estimates to differences in genotyping strategies and proportions of genotyping in these datasets. First, we computed mean and standard deviations for BW per SR for genotyped and ungenotyped males and females, and second, we plotted the distributions of standardized BW, which were obtained within SR, of genotyped and ungenotyped males and females. As will be shown in the Results section, females were preselected among the heavy individuals based on own BW, but this was less the case for males, and the proportion of genotyping was higher in females than in males. VC estimates were compared between A-AM and H-AM for the three groups (all data, males, and females), and related to the different genotyping strategies and proportions of genotyping used in each group. Significance of differences was tested by computing a *t* test for the difference between two means as $t = \left| {VC_{i} - VC_{j} } \right|/\sqrt {SE_{i}^{2} + SE_{j}^{2} }$. However, since females were genotyped both selectively and at a higher proportion, the comparison between males and females was not sufficient to separate the effects of selective genotyping and proportion of genotyping.

In the second set of analyses, we compared VC estimates from H-AM for different subsets of the data that were constructed to either enhance or mitigate the effects of selective genotyping and proportion of genotyping, and thus to better disentangle these two effects. Subset 1 increased the effect of selective genotyping in males, while Subsets 2 and 3 modified the proportion of genotyping by either removing genotyped or ungenotyped animals. These subsets were constructed as follows.

#### Subset 1

In Subset 1, selective genotyping was increased in males and proportion of genotyping was decreased, by using only the genotypes of the heavy males among all genotyped males (this was possible in males because both low-weight and high-weight males were genotyped). This made the genotyping strategy in males similar to the genotyping strategy in females, but with a lower proportion of genotyped animals. Genotyped males were ranked according to BW records, then genotypes from the heaviest 3000 individuals were kept to compute a new ${\mathbf{G}}_{{{\mathbf{m}}3{\mathbf{k}}}}$ matrix together with all the genotyped females, and the genotypes from the remaining genotyped males were removed in this subset. VC were estimated for the whole dataset, and for males only, using H-AM with the ${\mathbf{H}}$ matrix based on ${\mathbf{G}}_{{{\mathbf{m}}3{\mathbf{k}}}}$. All available phenotypes were used in the analyses, i.e. only part of the genotype information from the males was removed.

#### Subset 2

In Subset 2, the effect of selective genotyping was decreased in females by randomly removing genotypes of females. Instead of using all the 14,838 genotyped females, two random subsets of 8000 and 4000 individuals were sampled from genotyped females, and ${\mathbf{G}}_{{{\mathbf{f}}8{\mathbf{k}}}}$ and ${\mathbf{G}}_{{{\mathbf{f}}4{\mathbf{k}}}}$ were computed using genotypes from these two subsets together with all the genotyped males. In these subsets, females were selectively genotyped in a similar way as in the whole dataset, but the proportion of genotyping was decreased. VC were estimated for females only using H-AM with the ${\mathbf{H}}$ matrices based on ${\mathbf{G}}_{{{\mathbf{f}}8{\mathbf{k}}}}$ or ${\mathbf{G}}_{{{\mathbf{f}}4{\mathbf{k}}}}$. Phenotypes from all females were used in the analysis, i.e., only part of the genotype information from the females was removed.

#### Subset 3

In Subset 3, selective genotyping in males, as in Subset 1, and proportion of genotyping were increased simultaneously. This was achieved by using the same heaviest 3000 genotyped males from Subset 1, combined with decreasing the number of ungenotyped males by removing their phenotypes, so that, relatively, the proprortion of selectively genotyped males increased. To reach a proportion of genotyping of 30% in males, a random subset of 7000 ungenotyped males were sampled. Using phenotypes from these 7000 ungenotyped males together with the heaviest 3000 genotyped males in Subset 1, VC were estimated for males only using H-AM with the ${\mathbf{H}}$ matrix based on ${\mathbf{G}}_{{{\mathbf{m}}3{\mathbf{k}}}}$.

H-AM estimates in these three different subsets were compared to the H-AM estimates for the unedited data in the same group (all data, males, and females). Differences in VC estimates were tested with the same t-test as described above to test the difference between two means.

### Simulation study

After analyzing subsets of the commercial data, a population that mimicked the population structure and selection process of the broiler breeding program was simulated using the ADAM software [20]. The first objective of the simulation study was to verify the hypothesis that selective genotyping of a large proportion of birds caused bias in VC estimates from H-AM. The second objective was to show the existence of bias in VC estimates from A-AM after genomic selection was applied. Using simulated data makes it possible to modify genotyping strategy and selection scheme, and to remove the influence from potential other unknown factors in the commercial data, such that the conclusions based on simulated data are more straightforward. The term unbiased is used here in the frequentist sense as a property of maximum likelihood estimators to estimate, on average over replicated data samplings, the true value of a parameter [21].

The simulation was based on a historical population with a genome consisting of 40 K SNPs and 2 K quantitative trait loci (QTL), and the patterns of linkage disequilibrium (LD) decay along distances between each pair of SNPs were generated such that they were similar to that observed in the commercial data. Genetic effects were the sum of the QTL effects, which were sampled from a normal distribution. For both sexes, the total genetic variance in the base population was assumed to be 9644, and residual variance was assumed to be 24,798. Phenotypes were simulated as body weights (BW) which was the sum of additive genetic effects (${\text{u}}$) and residuals (${\text{e}}$). In each SR, 130 sires and 520 dams were mated to produce 5200 offspring. In total, 40 SR after the base population were simulated, and SR were divided into two periods depending on how the BV were computed. In SR1-20, parents were selected based on predicted BV by A-AM in all scenarios; in SR21-40, parents were selected based either on predicted BV by A-AM or on predicted BV by H-AM, using different genotyping strategies. In the simulation, VC estimates from the base population were used in the models to estimate BV. Three scenarios, which are summarized in Table 2, were simulated by varying the genotyping strategy and selection criteria.

**Table 2:** Models for breeding value prediction and genotyping strategies in the simulation of three scenarios

	SR1-20	SR21-40	Genotyping strategy in SR21-40
Scenario 1	A-AM	H-AM	Selective, 20%
Scenario 2	A-AM	H-AM	Random, 20%
Scenario 3	A-AM	A-AM	None

*SR* selection round, *A-AM* animal model with pedigree-based relationship matrix, *H-AM* Animal model with combined pedigree-based and genomic relationship matrix

#### Scenario 1

Scenario 1 simulated a population under genomic selection by H-AM with selective genotyping in SR21-40. Based on the ranking of BW within each SR, the 20% heaviest birds were selected to be genotyped, and the ${\mathbf{G}}$ matrix was computed based on these genotypes.

#### Scenario 2

Scenario 2 simulated a population under genomic selection by H-AM with random genotyping in SR21-40. A random subset of 20% birds were selected as the genotyped individuals in each SR, and the ${\mathbf{G}}$ matrix was computed based on these genotypes.

#### Scenario 3

Scenario 3 simulated a population with selection on predicted BV by A-AM for all SR1-40. i.e. no genomic information was used in the selection. However, the genotypes of all the birds were saved, and thus available for analyses using the H-AM models.

Scenarios 1 and 2 were designed to investigate the effects of genomic selection and different genotyping strategies on VC estimates, and to further test the hypothesis that selective genotyping causes bias in VC estimates from H-AM. Scenario 3 was used to check whether VC estimates were biased when using H-AM with selectively genotyped animals in the ${\mathbf{G}}$ matrix, even when the population was not under genomic selection.

For analysis of the simulated data, only the phenotypes from SR 30 to 40 were used, but with pedigree information going back to the base population. We did not expect that A-AM would correctly estimate the genetic variance in the base population from these analyses for two reasons: (1) selection before SR 30 cannot be accounted for, because the phenotypes on those generations were not included in the analysis, and (2) the simulated data are generated based on a finite locus model in which genetic variance can be lost due to fixation of QTL under selection. A-AM cannot correctly model this change of genetic variance, because it assumes an infinite locus model in which allele frequencies at underlying loci do not change. For these reasons, we expected the A-AM genetic variance estimates to be lower than those of the base population. Additional file 1: Figure S1 plots the genetic variance per generation, computed from the variances of true BV in each generation, and shows a reduction in genetic variance from the original base population variance of 9644 to an average of ~ 6000 in SR 30 to 40 that we used in our analysis. Thus, we compared the genetic variance estimates both to the genetic variance of the base population, and to the A-AM estimate in Scenario-3, and expected the latter to be a better benchmark for the expected VC estimates in this dataset.

Phenotypes, genotypes and true BV of all birds were saved for all scenarios. Therefore, VC can be estimated after simulation using different models and under different genotyping strategies. This includes scenarios in which genomic selection was based on selective genotyping, but estimation of VC used a random set of genotypes, or vice versa. Full pedigree, and phenotypes of birds in SR31-40, were used to estimate VC in both A-AM and H-AM. To investigate the effects of genotyping strategy and proportion of genotyping on VC estimates from H-AM, different proportions (10, 20 and 30%) of either a selected or random set of birds in SR31-40 were assumed to be genotyped, and these genotypes were used to construct the ${\mathbf{G}}$ matrix, and thus the ${\mathbf{H}}$ matrix for H-AM. For each scenario, three replicates were performed, and results are presented as the mean estimates over replicates and standard errors (SE) of the mean based on the standard deviation of the replicated estimates divided by the square root of the number of replicates. The estimated VC were compared to the variances of the base population and to A-AM estimates of Scenario 3 using a t-test as described above.

### Predictive ability, prediction accuracy and bias of predicted breeding values

In the commerial data, we evaluated predictive ability, computed as the correlation between predicted BV and phenotypes corrected for fixed effects (${\text{y}}_{\text{c}}$). The population was divided into training and validation data, in which all the birds from SR68-72 were grouped into the training set; full sibs from the same dam in SR73-77 were randomly divided into two subsets, one subset was merged to the training set and the other subset was assumed as the validation set; a bird without siblings was placed in the training set. The reason to define the validation group in this way was to equalize random maternal permanent environmental effects (${\mathbf{m}}$) between training and validation sets. However, with full-sibs of validation birds in the training data, the obtained accuracies will be higher than what would be achieved in reality with a forward prediction. Fixed effects were estimated using phenotypes of all birds in SR1-77. BV was predicted for birds in the validation set with own phenotypes masked as unknown, by the three following models: A-AM using VC estimates from A-AM; H-AM using VC estimates from A-AM; H-AM using VC estimates from H-AM.

For the simulated data, birds from SR31-35 were used as training set and birds from SR36-40 were used as validation set. Prediction accuracy was computed as the correlation between true BV and predicted BV. BV were predicted by H-AM using three different VC estimates, which were estimated by H-AM or A-AM, or were VC estimates of the base population. Prediction accuracy under different genotyping strategies with different proportions of genotyping were also compared between scenarios.

To evaluate “bias”, i.e. deflation or inflation in prediction of BV, corrected phenotypes (${\text{y}}_{\text{c}}$) were regressed on predicted BV for the commercial data, and true BV were regressed on predicted BV for the simulated data. The deviation of this regression coefficient from 1 indicates bias, with a regression coefficient smaller than 1 implying inflation in the prediction of BV, and a regression coefficient larger than 1 implying deflation in the prediction of BV.

## Results

### Commercial data

**Fig. 1:**
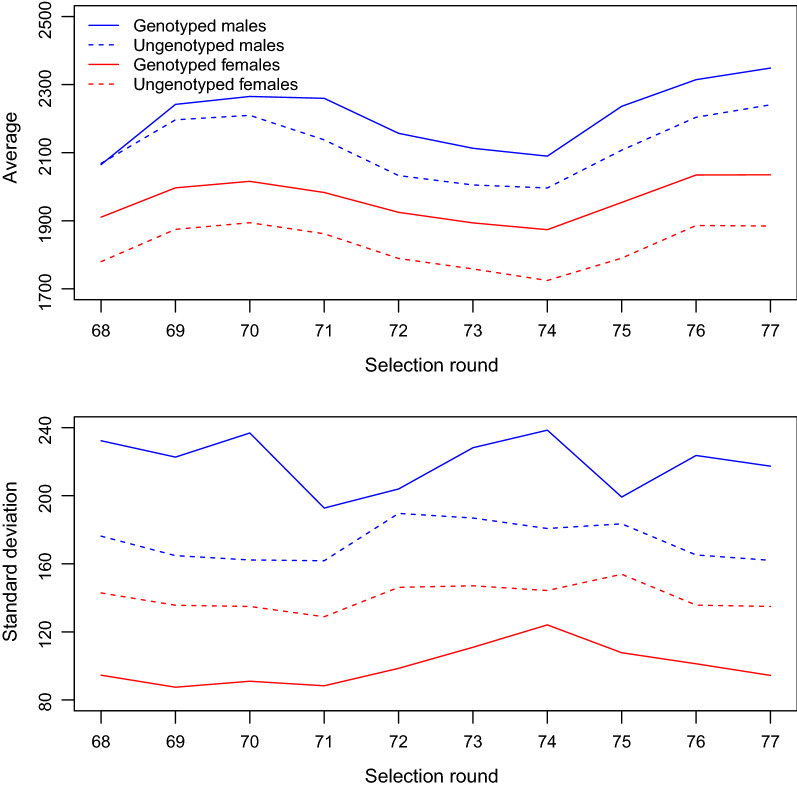
Mean and standard deviation of body weights per selection round (SR), for males and females and for genotyped and ungenotyped birds

**Fig. 2:**
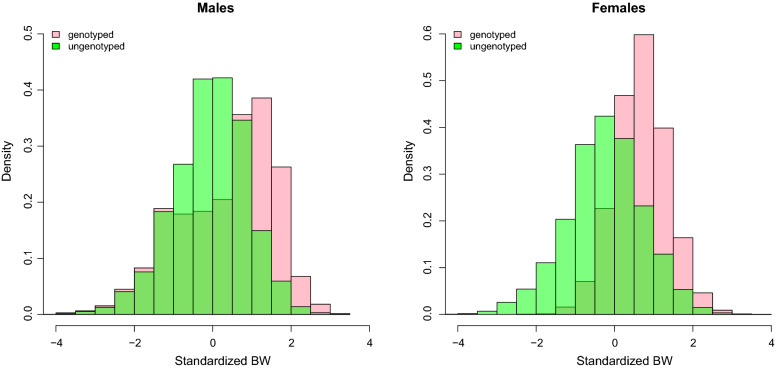
Histogram of standardized body weights (BW) for genotyped and ungenotyped males and females. Standardization was done within each selection round, by substracting the mean and dividing the standard deviation of body weights

The average and standard deviations of BW per SR for genotyped and ungenotyped males and females are in Fig. 1, and the distributions of standardized BW of genotyped and ungenotyped males and females are in Fig. 2. Generally, genotyped individuals were heavier than ungenotyped individuals, and this effect was strongest in females. However, variation in BW was larger for genotyped males than ungenotyped males, whereas it was smaller for genotyped females than ungenotyped females. This latter effect can be explained from Fig. 2, which shows that genotyped females are only heavy-weight individuals, whereas for males both heavy and light individuals were genotyped. In addition, the proportion of genotyped individuals was much higher in females than in males (Table 1).

The results from the first set of analyses, which analysed all data, males and females by A-AM and H-AM are in Table 3. They show that H-AM estimated a significantly larger genetic variance (over 2 times larger), and a significant smaller residual variance than A-AM. When analyzing males and females separately, A-AM estimates of the genetic variance did not differ significantly between males and females, and A-AM and H-AM estimates of the genetic variance did not differ significantly for males. However, for females, estimates of the genetic variance from H-AM were more than five times larger and highly significantly different from the A-AM estimates. Residual variances followed opposite patterns and were very similar (although only just significantly different) in males, but much smaller and highly significantly different in females for H-AM compared to A-AM. Hence, the difference in VC estimates between A-AM and H-AM observed in the whole dataset, seems to originate primarily from the females.

**Table 3:** Estimates of variance components (with SE in brackets) using the broiler data

Data	Model	$\hat{\sigma }_{a}^{2}$	$\hat{\sigma }_{m}^{2}$	$\hat{\sigma }_{e}^{2}$
All	A-AM	5007 (482.2)^*^	1414.5 (104.5)	17,062 (249.1)^*^
All	H-AM	12,498 (337.6)^*^	1269.8 (755.5)	13,775 (144.7)^*^
Males	A-AM	5208 (610.8)	1888.7 (152.7)	21,459 (329.5)^*^
Males	H-AM	6606 (385.8)	1920.8 (120.6)	20,567 (217.0)^*^
Females	A-AM	4653 (450.1)^*^	1117.1 (96.4)^*^	11,734 (233.1)^*^
Females	H-AM	23,524 (297.4)^*^	594.7 (56.3)^*^	4485 (80.3)^*^

$$\hat{\sigma }_{a}^{2}$$: genetic variance

$\hat{\sigma }_{m}^{2}$: variance of maternal permanent environmental effects

$$\hat{\sigma }_{e}^{2}$$: residual variance

*A-AM* animal model with pedigree-based relationship matrix, *H-AM* animal model with combined pedigree-based and genomic relationship matrix

^*^Significantly different comparing A-AM versus H-AM estimates within group All, Males or Females (P < 0.05)

In the second set of analyses on the commercial data, we investigated the effects of selective genotyping and proportion of genotyping on VC estimates in H-AM by analyzing three subsets of the commercial data. The results are shown in Table 4. In Subset 1, only the genotypes of heavy males and of all available females, were used to construct ${\mathbf{G}}$. For all data, this increased $\hat{\sigma }_{a}^{2}$ to 29,054 (297.4) and decreased $\hat{\sigma }_{e}^{2}$ to 6719 (104.5), which is significantly more compared to the H-AM analysis for all data in Table 3. For the males only, $\hat{\sigma }_{a}^{2}$ was estimated at 6341 (514.4), which is not significantly different from 6606 (385.8) when using the unedited male dataset (Table 3). In Subset 2, the proportion of genotyped females was decreased, resulting in a significant decrease in $\hat{\sigma }_{a}^{2}$ to 14,651 (409.9) when using 8000 genotyped females and even more to 6020 (434.0) when using 4000 genotyped females, compared to 23,524 (297.4) when using all genotyped females. The H-AM VC estimates of genetic variance for females by using 4000 genotyped females were no longer significantly different from the H-AM estimates in males, and were also close to the VC estimates from A-AM for females in Table 3. In Subset 3, the proportion of selectively genotyped males increased to 30%, and $\hat{\sigma }_{a}^{2}$ when analysing males only increased to 31,722 (1824.4), which is a significant increase compared to the results in Table 3. Taken together, these results show that if the proportion of selectively genotyped birds was small (Subset 1 for males only and Subset 2 with 4000 selectively genotyped females), VC from A-AM and H-AM did not differ significantly, but when a large proportion of selectively genotyped birds was used to construct ${\mathbf{G}}$ (Subset 1 for all data and Subset 3 for males), genetic variance estimates from H-AM were significantly larger than those from A-AM.

**Table 4:** Estimates of variance components (SE) from H-AM using three subsets of the real data

Subset^a^	Data	$\hat{\sigma }_{a}^{2}$	$\hat{\sigma }_{m}^{2}$	$\hat{\sigma }_{e}^{2}$	Number of genotyped birds (genotyping proportion %)
1	All	29,054 (297.4)^*^	643.0 (56.3)^*^	6719 (104.5)^*^	17,838 (16.43)
	Male	6341 (514.4)	1872.6 (128.6)	20,824 (281.3)	3000 (5.57)
2	Female	14,651 (409.9)^*^	755.5 (72.3)	7065 (176.8)^*^	8000 (14.62)
	Female	6020 (434.0)	1068.9 (80.4)^*^	11,011 (225.0)^*^	4000 (7.31)
3	Male	31,722 (1824.4)^*^	1977.1 (401.9)	10,979 (691.2)^*^	3000 (30)

$$\hat{\sigma }_{a}^{2}$$: genetic variance

$\hat{\sigma }_{m}^{2}$: variance of maternal permanent environmental effects

$$\hat{\sigma }_{e}^{2}$$: residual variance

*H-AM* Animal model with combined pedigree-based and genomic relationship matrix

^*^Significantly different comparing against the H-AM estimates for the same group (All, Male, Female) in Table 3 (P < 0.05)

^a^For description of the Subsets see the main text

### Simulation study

The estimates of VC from H-AM and A-AM in the three scenarios are summarized in Table 5, which also contains the average value of VC estimates and their average SE across the three replicates. VC estimates from different scenarios and models were compared to the variances for the base population, and to A-AM estimates from Scenario 3 (population under A-AM selection).

**Table 5:** Average VC estimates across replicates (averaged standard error) in the simulation study

Selection method in simulation	Analysis model	Analysis genotyping strategy and proportion (%)		$\hat{\sigma }_{a}^{2}$ (SE)	$\hat{\sigma }_{e}^{2}$ (SE)
Scenario 1: H-AM with 20% selective genotyping in SR21-40	H-AM	Selective	10	13,358 (635)^ab^	22,358 (324)^ab^
			20	40,597 (729)^ab^	11,904 (243)^ab^
			30	55,051 (639)^ab^	8695 (136)^ab^
		Random	10	9265 (469)	24,231 (270)^ab^
			20	8967 (440)	24,382 (243)
			30	8873 (421)	24,461 (223)
	A-AM			11,475 (544)^ab^	23,148 (312)^56^
Scenario 2: H-AM with 20% random genotyping in SR21-40	H-AM	Selective	10	13,370 (641)^ab^	22,379 (328)^ab^
			20	43,302 (697)^ab^	10,911 (223)^ab^
			30	55,735 (635)^ab^	8462 (133)^ab^
		Random	10	8645 (449)^b^	24,581 (265)
			20	8408 (423)^b^	24,729 (241)
			30	8507 (410)^b^	24,689 (222)
	A-AM			9598 (477)^a^	24,125 (289)^ab^
Scenario 3: A-AM in SR1-40	H-AM	Selective	10	11,402 (561)^ab^	23,346 (305)^ab^
			20	37,343 (735)^ab^	12,947 (260)^ab^
			30	53,307 (633)^ab^	9037 (142)^ab^
		Random	10	8347 (441)^b^	24,825 (267)
			20	8427 (425)^b^	24,789 (244)
			30	8470 (404)^b^	24,740 (223)
	A-AM			8206 (433)^b^	24,902 (278)

$\hat{\sigma }_{a}^{2}$: genetic variance; $\hat{\sigma }_{e}^{2}$: residual variance

*H-AM* animal model with combined pedigree-based and genomic relationship matrix, *A-AM* animal model with pedigree-based relationship matrix

^a^Significantly different from the A-AM variance in Scenario 3 (P < 0.05)

^b^Significantly different from the base-population variances (P < 0.05)

For H-AM, different genotyping strategies were compared by selecting three proportions (10, 20 and 30%) of either preselected or random birds to be genotyped and using genotypes of selected birds to compute the ${\mathbf{G}}$ matrix. When using genotypes from preselected heavy birds in the H-AM analysis, estimates of the genetic variance ($\hat{\sigma }_{a}^{2}$) were all significantly larger than both the variance of the base population and the A-AM estimates from Scenario 3, and the over-estimation increased as proportion of genotyping increased. This is also true when the population was selected by using A-AM. In contrast to $\hat{\sigma }_{a}^{2}$, the estimated residual variance $\hat{\sigma }_{e}^{2}$ was significantly underestimated in all scenarios using selective genotyping in the H-AM analysis. However, when using genotypes from a random subset of birds in H-AM, VC estimates did not differ significantly from the A-AM estimates of Scenario 3 for all scenarios, but Scenarios 2 and 3 underestimate significantly the variance of the base population.

The results of the analyses with A-AM in populations under genomic selection (Scenarios 1 and 2) showed that $\hat{\sigma }_{a}^{2}$ was significantly larger than the variance of the base-population in Scenario 1, and was significantly larger than the A-AM estimate from Scenario 3, in Scenarios 1 and 2. The A-AM estimate in Scenario 3 was significantly lower than the base population $\sigma_{{a_{0} }}^{2}$ (t-value = 3.32).

### Predictive ability, prediction accuracy and bias of predictions

For the commercial data, predictive ability and bias of predictions were compared among the three models: A-AM, H-AM and H-AM (VC-A), which is H-AM using VC estimates from A-AM. Table 6 shows that for both males and females, H-AM (VC-A) and A-AM had the highest and lowest predictive ability, respectively. H-AM had a higher predictive ability for genotyped birds than for ungenotyped birds, while A-AM and H-AM (VC-A) had a lower predictive ability for genotyped birds than for ungenotyped birds.

**Table 6:** Predictive ability^a^ and bias of predictions^b^ in the commercial data

	Model	Male		Female	
		Genotyped	Ungenotyped	Genotyped	Ungenotyped
Predictive ability	A-AM	0.15	0.21	0.25	0.26
	H-AM	0.31	0.22	0.40	0.28
	H-AM (VC-A)	0.40	0.42	0.44	0.49
Regression coefficient^c^	A-AM	0.93	0.96	0.89	0.95
	H-AM	1.01	0.98	0.83	0.77
	H-AM (VC-A)	0.99	0.90	0.98	0.89

*A-AM* animal model with pedigree-based relationship matrix using VC estimated from A-AM, *H-AM* animal model with combined pedigree-based and genomic relationship matrix using VC estimated from H-AM, *H-AM(VC-A)* animal model with combined pedigree-based and genomic relationship matrix using VC estimated from A-AM

^a^Predictive ability is correlation between predicted breeding value and corrected phenotype

^b^Bias of predicted breeding value is measured by the regression coefficients of predicted breeding values on corrected phenotypes, deviation from unity indicates a bias (inflation or deflation) in breeding values

^c^Regression coefficients of predicted breeding values on corrected phenotypes

Generally, bias of predictions was smaller for males than for females, and all biases indicate inflation of predictions. Bias was largest for females with H-AM, but males were close to unbiased with H-AM. Furthermore, levels of bias differed between genotyped and ungenotyped birds, but this was not consistent across models: bias was larger for genotyped than for ungenotyped birds (both in males and females) with A-AM, but bias was smaller (close to absent) for genotyped than ungenotyped birds with H-AM (VC-A). There was no clear connection between predictive ability and bias of predictions.

For the simulated data, prediction accuracy and inflation were compared between three H-AM using different VC: VC estimated in H-AM or A-AM, or VC of the base population (Tables 7, 8). The results show that when using ${\mathbf{H}}$ based on selective genotyping, the accuracy was lower for genotyped and ungenotyped birds when using VC from H-AM than from A-AM and when using VC from the base population. Within the same scenario, prediction accuracies were higher for random genotyping than for selective genotyping when comparing within genotyped or ungenotyped birds and for the same proportion of genotyping. When genotyped and ungenotyped birds were compared, prediction accuracy was higher for genotyped than for ungenotyped birds in all scenarios and for all genotyping strategies. The bias was particularly strong for the ungenotyped group when using VC from H-AM with selective genotyping and a high proportion of genotyping (20 and 30%).

**Table 7:** Average prediction accuracy^a^ using H-AM with different variance components (VC) in the simulated data

Scenario	Genotyping strategy (%)		VC-H		VC-A		VC-bp	
			Genotyped	Ungenotyped	Genotyped	Ungenontyped	Genotyped	Ungenotyped
1	Selective	10	0.78	0.54	0.78	0.54	0.78	0.54
		20	0.73	0.40	0.83	0.53	0.83	0.54
		30	0.70	0.35	0.84	0.52	0.84	0.53
	Random	10	0.81	0.57	0.81	0.57	0.81	0.57
		20	0.86	0.58	0.86	0.57	0.86	0.58
		30	0.88	0.58	0.88	0.58	0.88	0.58
2	Selective	10	0.78	0.54	0.79	0.56	0.79	0.56
		20	0.72	0.40	0.83	0.55	0.82	0.55
		30	0.70	0.36	0.84	0.54	0.84	0.54
	Random	10	0.83	0.59	0.83	0.59	0.83	0.59
		20	0.87	0.59	0.87	0.59	0.87	0.59
		30	0.88	0.60	0.88	0.60	0.88	0.60
3	Selective	10	0.80	0.59	0.80	0.60	0.80	0.60
		20	0.75	0.45	0.83	0.59	0.83	0.59
		30	0.72	0.39	0.85	0.58	0.85	0.58
	Random	10	0.84	0.62	0.84	0.62	0.84	0.62
		20	0.87	0.63	0.87	0.63	0.87	0.63
		30	0.89	0.63	0.89	0.63	0.89	0.63

*H-AM* animal model with combined pedigree-based and genomic relationship matrix, *VC-H* VC estimates from H-AM, *VC-A* VC estimates from A-AM (animal model with pedigree-based relationship matrix), *VC-bp* VC of the base population

^a^Prediction accuracy is correlation between true and predicted breeding value

**Table 8:** Average bias of predictions^a^ from H-AM with different variance components (VC) in the simulated data

Scenario	Genotyping strategy (%)		VC-H		VC-A		VC-bp	
			Genotyped	Ungenotyped	Genotyped	Ungenotyped	Genotyped	Ungenotyped
1	Selective	10	0.96	0.76	0.96	0.82	0.96	0.88
		20	0.93	0.33	0.99	0.85	0.99	0.91
		30	0.97	0.26	1.04	0.88	1.04	0.93
	Random	10	0.91	0.87	0.88	0.79	0.91	0.86
		20	0.94	0.89	0.91	0.80	0.93	0.87
		30	0.95	0.90	0.93	0.81	0.95	0.88
2	Selective	10	1.01	0.80	1.02	0.92	1.02	0.93
		20	0.98	0.31	1.06	0.95	1.06	0.96
		30	1.01	0.27	1.10	0.98	1.10	0.98
	Random	10	0.96	0.95	0.94	0.91	0.95	0.92
		20	0.99	0.96	0.97	0.91	0.97	0.92
		30	0.98	0.96	0.97	0.92	0.97	0.93
3	Selective	10	1.01	0.91	1.03	1.00	1.02	0.97
		20	0.99	0.41	1.07	1.02	1.06	0.99
		30	1.06	0.31	1.11	1.03	1.11	1.01
	Random	10	0.99	0.99	0.99	1.00	0.97	0.95
		20	0.99	0.99	0.99	1.00	0.98	0.96
		30	0.99	1.00	0.99	1.00	0.98	0.96

*H-AM* animal model with combined pedigree-based and genomic relationship matrix, *VC-H* VC estimates from H-AM, *VC-A* VC estimates from A-AM (animal model with pedigree-based relationship matrix), *VC-bp* VC of the base population

^a^Bias of predicted breeding values is presented as the regression coefficient of the true breeding values on the predicted breeding values; deviation from 1 implies bias, regression coefficients lower than 1 imply inflation in the predictions

## Discussion

The first and main aim of our study was to show that there is a (large) overestimation of the genetic variance by H-AM with selective genotyping at a sufficienly high proportion of genotyping. Using the broiler dataset, we showed that the proportion of genotyping is higher for females than for males, and genotyping is performed only on heavy animals (Table 1, Figs. 1, 2). The estimates of genetic variance by H-AM and A-AM were similar and did not differ significantly for males, whereas for females the H-AM estimate of genetic variance was more than 5 times larger than the A-AM estimate, which represents a highly significant difference (Table 3). This suggests that the extreme high estimate of the genetic variance by H-AM for females is due to the females having a higher proportion and more selectively genotyped individuals. Further editing of the data showed that we could increase or decrease H-AM estimates of genetic variance by modifying the genotyping stratetegy and proportion of genotyping, which corroborates the connection between large estimates of genetic variance by H-AM and a high proportion of selectively genotyped individuals in the analysis (Table 4). In the simulation study, we could replicate these effects and confirmed that H-AM estimates of genetic variance could be highly biased upwards under selective genotyping: we simulated a genetic variance in the base population of 9644, but with a high proportion (30%) of selective genotyping, H-AM obtained estimates of genetic variance larger than 50,000 (Table 5). These findings based on both real and simulated data clearly show that the estimation of VC by H-AM is very sensitive to genotyping strategy and proportion of genotyping, with large overestimates of genetic variance under selective genotyping when the proportion of genotyped individuals is high.

Few studies have used H-AM for VC estimation. One study estimated VC in pigs [22] and reported similar heritabilities for three of the traits studied, and an increased heritability for a fourth trait (from 0.29 (SE 0.04) using A-AM to 0.36 (SE 0.03) using H-AM). Selection and genotyping strategies are not described in [22], but if genotyping was (close to) random with respect to the first three traits, and selective based on the fourth trait, this result could support our findings.

H-AM is A-AM with genotype data added for part of the individuals, and if the proportion of genotyping is low, it is logical that H-AM and A-AM lead to similar results and that the amount of bias in H-AM from selective genotyping depends on the proportion of genotyped animals. We were able to show this effect clearly: with the commercial data, we created an edited dataset with 5.57% of selectively genotyped males, and observed no difference between the variance estimates obtained with H-AM and A-AM (Table 4). In the simulated datasets, at a proportion of genotyping of 10%, we found a significant but still modest upwards bias in H-AM (Table 5). Hence, up to a proportion of 10% of genotyped individuals, overestimation of genetic variance by H-AM under selective genotyping remained small. From a proportion of ~ 20% selective genotyping, overestimation of genetic variance by H-AM became large.

Based on our simulation studies, we could separate two effects related to selective genotyping: (1) the effect of using selective genotyping in the genomic selection; and (2) the effect of using a selective sample of genotyped individuals in the ${\mathbf{H}}$ matrix. When we analyzed Scenario 1, which was under genomic selection with selective genotyping, and used a set of random genotypes to construct ${\mathbf{H}}$, no strong biases were observed (Table 5). Hence, the bias does not arise from using genomic selection with selective genotyping. However, in Scenario 2, which was under genomic selection with random genotyping, and in Scenario 3, which did not use genomic selection at all, we obtained highly biased estimates from H-AM when using a selective sample of genotypes in the ${\mathbf{H}}$ matrix (Table 5). Hence, the bias in H-AM is uniquely associated with the use of a selective group of genotypes in the ${\mathbf{H}}$ matrix, irrespective of the underlying selection process. An associate editor of this journal suggested that the cause of the bias in H-AM under selective genotyping is related to the ${\mathbf{G}}$-matrix not properly modelling the “segmental nature of inheritance of DNA” [23], i.e., the standard procedures to construct ${\mathbf{G}}$ do not account for the physical linkage between markers, and the recombination events that create shared identical by descent (IBD) genomic segments between parents and progeny. This suggests that the problems, which we observed, could be repaired by constructing estimates of genomic relationships that are based on tracing segregation and IBD based on pedigree data. Other studies have addressed the issue that the estimators of genetic variance based on ${\mathbf{G}}$ (or ${\mathbf{H}}$) ignore LD between QTL, which can cause both underestimation [24] and overestimation [25] of genetic variance, depending on the predominant sign of the LD between QTL. Under selection, this LD should be negative, leading to overestimation [25]. In our results, we did not see overestimation with random genotyping, but only with selective genotyping. This could imply that selection in itself was not strong enough to cause significant LD between QTL, but with selective genotyping such an effect may have been induced and led to overestimation of genetic variance.

The second objective of this study was to investigate the hypothesis that A-AM could provide biased estimates of genetic variance in populations under genomic selection, and whether H-AM with random genotyping can be an alternative approach. This is based on the simulation study for which we know the underlying QTL and genetic variances. One issue in interpreting the results from the simulation study is that, as described in Methods, we expect genetic variance estimates to be lower than that of the original base population because (1) the selection history up to SR 30 was hidden by not including phenotypes from these SR in the analysis; and (2) we used a finite-locus model to simulate genomic selection causing the genetic variance to decrease across generations due to the change in allele frequencies at the QTL. Thus, we compared VC estimates both to the original base population variance (9644), and to the A-AM estimate for populations under A-AM selection (Scenario 3), which estimated genetic variance at 8206 (Table 5).

The results with A-AM in populations under genomic selection (Scenario 1 and 2) showed that A-AM estimates were clearly biased upwards in Scenario 1, but in Scenario 2 the conclusion is ambiguous because the genetic variance estimate did not differ significantly from the variance of the base population, but did differ significantly from the A-AM estimate in the population without genomic selection (Table 5). The difference between Scenarios 1 and 2 can be explained by genomic selection being more efficient in Scenario 1 (based on selective genotyping) than in Scenario 2 (based on random genotyping). Hence, we conclude that VC estimates from A-AM are indeed biased for populations under genomic selection, but the bias may not be clearly visible when genomic selection is less efficient. The upward bias in estimates of genetic variance from A-AM can be explained by genomic selection being more efficient than what A-AM can explain based on information from phenotypes and pedigree. The selection response in populations under genomic selection is likely higher than what the phenotypic selection differential can explain, and as a consequence A-AM needs to inflate its heritability in order to explain the extra selection response due to exploitation of genomic information. Hence, the common assumption that A-AM can give unbiased VC estimates is no longer met after implementing genomic selection, because A-AM cannot capture the effects of selection based on genomic information. In the long term, breeding programs must not overlook the bias in VC estimates from A-AM, and alternative models and methods to estimate VC in populations under genomic selection should be developed.

We verified whether H-AM with random genotyping could be an alternative for estimating genetic variance in populations under genomic selection and we found that the estimates were significantly smaller than the variance of the base population for Scenarios 2 and 3, but for all scenarios they did not differ from the Scenario 3 A-AM estimate for a population that was not under genomic selection. If one assumes that analysis of this data should retreive the variance of the base population, these lower estimates are difficult to explain. However, if one assumes, for the reasons explained above, that analysis of this data should result in estimates of genetic variance smaller than the variance of the base population, then the Scenario 3 A-AM estimate and all H-AM estimates with random genotyping fit this expectation. We concluded that the results from H-AM with random genotyping remain ambiguous, but suggest that H-AM with random genotyping may be more appropriate than A-AM for the estimation of genetic variance in populations under genomic selection.

As already noted above, H-AM with a low proportion of genotyping should perform similarly to A-AM. Since we have established that A-AM overestimates genetic variances for populations under genomic selection, at least clearly in Scenario 1 with strong genomic selection, then H-AM with a low proportion of genotyping should also overestimate genetic variances. Indeed, in Scenario 1 estimates of genetic variance from H-AM with random genotyping tend to increase when the proportion of genotyping decreases. This trend is small and not significant, but in line with the overestimation by A-AM. We also observed that H-AM with 10% random genotyping is sufficient to make a significant downward adjustment on the overestimate of A-AM in the scenario with genomic selection using selective genotyping (in Scenario 1, the H-AM estimate of 9265 ± 469 differs significantly from the A-AM estimate of 11,475 ± 544, t-value = 3.08). The results from using H-AM with random genotyping in the population under genomic selection with selective genotyping (Scenario 1), suggest that a dual genotyping strategy could be used: selective genotyping for estimation of BV using H-AM, which is known to lead to high accuracy, and a random genotyping for estimation of VC.

Use of inappropriate VC can generate bias in predicted BV [26], with “bias” here referring to bias in the classical animal breeding literature on unbiased prediction [27]. The use of unbiased predictors is a cornerstone in animal breeding, as it allows comparison of BV and selection across groups for which different amounts of information are available [27]. The results from the simulation study clearly showed that, in several cases, the variance of predicted BV was too large, because the regression coefficients were mostly smaller than 1 in populations under genomic selection (Scenarios 1 and 2 in Table 8). When using VC estimated from H-AM with a high proportion of genotyping (20 and 30%) among the preselected heavy birds, the regression coefficients for ungenotyped birds ranged only from 0.26 to 0.31, whereas the regression coeffients for genotyped birds were higher than 0.9. This means that predicted BV for ungenotyped birds were severely inflated, which would introduce suboptimal selection across genotyped and ungenotyped birds. However, when using VC from A-AM or base population parameters, or when using random genotyping, the regression coefficients were much closer to 1 and more similar between genotyped and ungenotyped birds. The inflation in predicted BV reflected the bias in VC estimates which was used in MME to predict BV. In the near future, as breeding programs tend to genotype more individuals, the genotyping strategy should be considered not only for maximizing genetic gain, but also for ensuring unbiased VC estimates in order to predict BV without (different) biases for genotyped and ungenotyped animals.

Our findings imply that populations under genomic selection need a proper strategy to collect genotypes, which should ensure that the current statistical models can provide unbiased VC estimates and thus unbiased prediction of BV. We showed that genotyping a high proportion of heavy birds in broiler breeding caused strong overestimation of the genetic variance, which also leads to bias in predicted BV. In addition, we showed that A-AM also overestimates the genetic variance in populations under genomic selection, although in our results this was clearly significant only in the scenario with the strongest genomic selection. Finally, our results suggest that H-AM with random genotyping could provide plausible estimates of VC. As in one of our scenarios, it is possible to combine selective genotyping for the purpose of selecting breeding animals, with random genotyping for the purpose of estimating VC. In addition, other selective genotyping strategies besides random genotyping have been considered in the literature, for instance genotyping both low and high extreme phenotypes was described for prediction of BV by [14, 15]. It could be interesting to investigate these alternative genotyping strategies also for estimating VC. Without better models, we recommend to avoid the use of selectively genotyped individuals for the estimation of VC using H-AM.

## Conclusions

In this study, we showed that estimates of genetic variance from a single-step animal model, in which genomic and pedigre relationships are combined (H-AM), can be severely overestimated when using selective genotyping at a high proportion. We found that, for populations under genomic selection, genetic variances estimated from the pedigree-based animal model (A-AM) were also biased. This is due to the inability of A-AM to capture the effect of genomic selection. When we used a random genotyping strategy, genetic variance estimates from H-AM were close to expected values. In addition, we demonstrated that predicted breeding values were biased when using inappropriate variance estimates, and more so for ungenotyped than for genotyped animals, which would lead to suboptimal selection across genotyped and ungenotyped animals. Therefore, we recommend to ensure that individuals from the whole distribution of phenotypes are genotyped.

## Supplementary information

**Additional file 1: Figure S1.** Genetic variance by generation in the simulated data computed from the variance of true breeding values in each generation.

## Data Availability

The commercial dataset analyzed in this study is available on request from the corresponding author with permission from Cobb-Vantress Inc. The simulated datasets generated are publicly available from the corresponding author on request.
