# Supplementary figures and images for: Effect of genomic selection and genotyping strategy on estimation of variance components in animal models using different relationship matrices
Source: Genet Sel Evol. 2020 Jun 11;52:31. doi: 10.1186/s12711-020-00550-w (PMC7291515; doi:10.1186/s12711-020-00550-w)

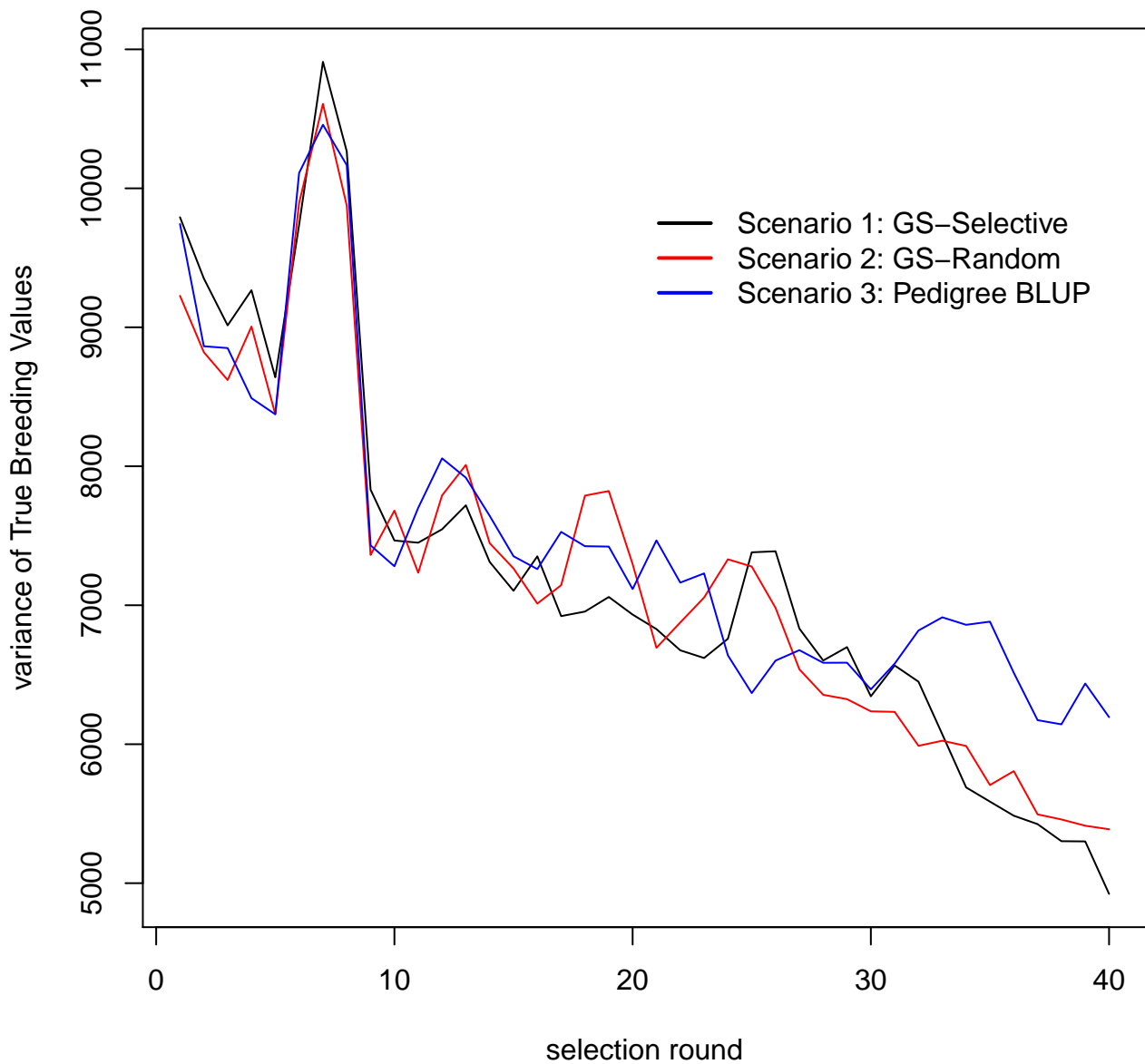

Supplement: Supplementary file 1 — Additional file 1: Figure S1. Genetic variance by generation in the simulated data computed from the variance of true breeding values in each generation. [file 12711_2020_550_MOESM1_ESM.pdf]
